# Matrix compliance and the regulation of cytokinesis

**DOI:** 10.1242/bio.011825

**Published:** 2015-05-22

**Authors:** Savitha Sambandamoorthy, Shomita Mathew-Steiner, Scott Varney, Jonathan M. Zuidema, Ryan J. Gilbert, Livingston Van De Water, Susan E. LaFlamme

**Affiliations:** 1 Center for Cell Biology and Cancer Research, Albany Medical College, Albany, NY 12208, USA; 2 Department of Biomedical Engineering, Rensselaer Polytechnic Institute, Troy, NY 12180, USA

**Keywords:** Integrins, Matrix compliance, Cytokinesis

## Abstract

Integrin-mediated cell adhesion to the ECM regulates many physiological processes in part by controlling cell proliferation. It is well established that many normal cells require integrin-mediated adhesion to enter S phase of the cell cycle. Recent evidence indicates that integrins also regulate cytokinesis. Mechanical properties of the ECM can dictate entry into S phase; however, it is not known whether they also can affect the successful completion of cell division. To address this issue, we modulated substrate compliance using fibronectin-coated acrylamide-based hydrogels. Soft and hard substrates were generated with approximate elastic moduli of 1600 and 34,000 Pascals (Pa) respectively. Our results indicate that dermal fibroblasts successfully complete cytokinesis on hard substrates, whereas on soft substrates, a significant number fail and become binucleated. Cytokinesis failure occurs at a step following the formation of the intercellular bridge connecting presumptive daughter cells, suggesting a defect in abscission. Like dermal fibroblasts, mesenchymal stem cells require cell-matrix adhesion for successful cytokinesis. However, in contrast to dermal fibroblasts, they are able to complete cytokinesis on both hard and soft substrates. These results indicate that matrix stiffness regulates the successful completion of cytokinesis, and does so in a cell-type specific manner. To our knowledge, our study is the first to demonstrate that matrix stiffness can affect cytokinesis. Understanding the cell-type specific contribution of matrix compliance to the regulation of cytokinesis will provide new insights important for development, as well as tissue homeostasis and regeneration.

## INTRODUCTION

Proliferation of adherent mammalian cells is tightly regulated by a number of factors including cell adhesion to the extracellular matrix (ECM) and the binding of soluble growth factors to their receptors (Assoian and Schwartz, 2001; Schwartz and Assoian, 2001). Integrins are the major family of receptors that mediate cell-ECM interaction (Hynes, 2002). Integrins coordinate with growth factor receptors to control entry into S phase (Assoian and Schwartz, 2001; Schwartz and Assoian, 2001). Accumulating evidence indicates that integrins can also regulate mitotic events, such as spindle positioning, orientation, and bipolarity (LaFlamme et al., 2008 180; Toyoshima and Nishida, 2007). Studies from our lab and others have demonstrated that integrins can also promote the successful completion of cytokinesis (Aszodi et al., 2003; Kittler et al., 2007; Mathew et al., 2014; Pellinen et al., 2008; Reverte et al., 2006; Thullberg et al., 2007).

Mechanical properties of the ECM impact cell behavior; many types of cells can sense and respond to the stiffness of the ECM. In the past two decades, immense progress has been made in deciphering the role of mechanical signaling in regulating cell morphology, cell migration, ECM remodeling, apoptosis, entry into S phase, and differentiation (Bae et al., 2014; Engler et al., 2006; Klein et al., 2009; Pelham and Wang, 1997; Wang et al., 2000; Yeung et al., 2005). However, the contribution of matrix compliance to the regulation of cytokinesis has not been analyzed until this current study.

Cytokinesis is the last step in cell division and begins with the assembly of the contractile ring (Green et al., 2012; White and Glotzer, 2012). Furrow ingression is driven by the constriction of the contractile ring, as daughter chromosomes are separated by the action of the mitotic spindle. After ingression is completed, daughter cells are connected by an intercellular bridge, which is also referred to as the midbody. The midbody serves as a platform for the recruitment of machinery that remodels the plasma membrane and cytoskeleton in preparation for abscission (Agromayor and Martin-Serrano, 2013; Guizetti and Gerlich, 2010; Schiel and Prekeris, 2013). The abscission machinery, including the ESCRT complex, is assembled at the midbody in a highly regulated stepwise process (Agromayor and Martin-Serrano, 2013; Guizetti and Gerlich, 2010; Schiel and Prekeris, 2013), and mediates the separation of daughter cells by fission of the midbody. Previous studies from our laboratory have implicated integrin signaling in the regulation of abscission (Mathew et al., 2014).

Microscopic analysis of cytokinesis of mammalian cells in 2D culture has shown that daughter cells migrate apart as cytokinesis nears completion resulting in forces pulling on the intercellular bridge, which has lead to the suggestion that these forces promote abscission, perhaps by thinning the bridge (Böttcher et al., 2009; Burton and Taylor, 1997). However, a recent study has shown that these forces on the intercellular bridge can delay abscission, at least in some instances (Lafaurie-Janvore et al., 2013). It is possible that tractional forces play complex roles, perhaps exerting opposing effects at different steps in the assembly of the abscission machinery. Alternatively, tractional forces may exhibit cell type–specific functions in regulating the completion of cytokinesis. Since the generation of tractional force is related to the stiffness of the matrix, we examined the effects of matrix compliance on the successful completion of cytokinesis. Compliance was modulated using fibronectin-coated acrylamide-based hydrogels. Our results indicate a cell type-specific reliance on matrix stiffness for the successful completion of cytokinesis.

## RESULTS

### Cell-matrix adhesion and β1 integrins promote the successful completion of cytokinesis in primary human dermal fibroblasts (HDFs)

**Fig. 1.**
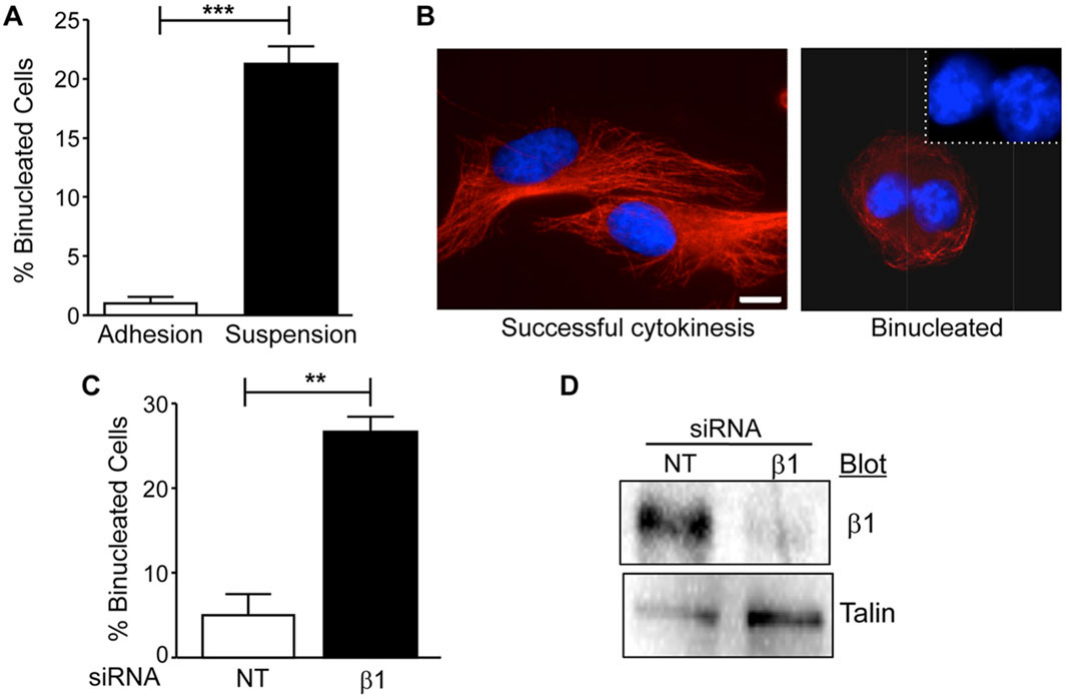
**Cell-matrix adhesion and β1 integrin expression promotes cytokinesis in primary human dermal fibroblasts (HDFs).** (A,B) Mitotic HDFs were collected by the shake-off method and replated onto either fibronectin-coated coverslips (adhesion) or poly-HEMA coated dishes (suspension) and incubated at 37°C for 5 h. Cells were fixed and stained for α-tubulin (red) and nuclei (blue) as described in Materials and Methods and then analyzed for binucleated cells. (A) Plotted is the mean percentage of binucleated cells±s.e.m. from three independent experiments in which more than fifty cells were counted per condition for each experiment (***P<0.0005). (B) Shown are representative images of cells that completed cytokinesis (adhesion) or failed cytokinesis/binucleated (suspension). Inset: magnified view of nuclei. Bar, 25 µm. (C,D) HDFs were transfected with 20 nM non-targeting (NT) siRNA or integrin β1 siRNA. (C) Plotted is the mean percentage of binucleated cells in non-targeting (NT) or β1 siRNA treated HDFs±s.e.m. from three independent experiments in which more than seventy-five cells were counted per condition for each experiment (**P<0.005). (D) The inhibition of β1 integrin expression was confirmed by western blotting. Blots were reprobed for talin as an internal control.

To examine the role of matrix compliance in the regulation of cytokinesis, we used primary human dermal fibroblasts (HDFs), as these cells are known to develop tension and pull against their ECM (Hinz, 2010). We began our study by confirming that cell-matrix adhesion promotes cytokinesis in HDFs. To assay the requirement for cell-matrix adhesion, mitotic HDFs were collected and replated either on polyHEMA to block adhesion or on fibronectin as a positive control. Cells were then incubated for 5 h at 37°C and analyzed for completion of cytokinesis. The results indicate approximately 20% of HDFs fail cytokinesis in the absence of cell-matrix adhesion, whereas less than 2% fail cytokinesis when adhered to fibronectin (Fig. 1A-B). These results are in agreement with previously published studies (Thullberg et al., 2007). To determine whether integrin-mediated adhesion was required to promote cytokinesis in HDFs, we inhibited the expression of β1 integrins using small interfering RNA (siRNA). HDFs were transfected with control or β1 siRNA and then analyzed for binucleation after 72 h. The results showed that approximately 26% of cells treated with siRNA targeting β1 integrins were binucleated (Fig. 1C-D). Thus, β1 integrins promote successful cytokinesis in HDFs.

### Compliance and fibronectin coating of hard and soft hydrogels

**Fig. 2.**
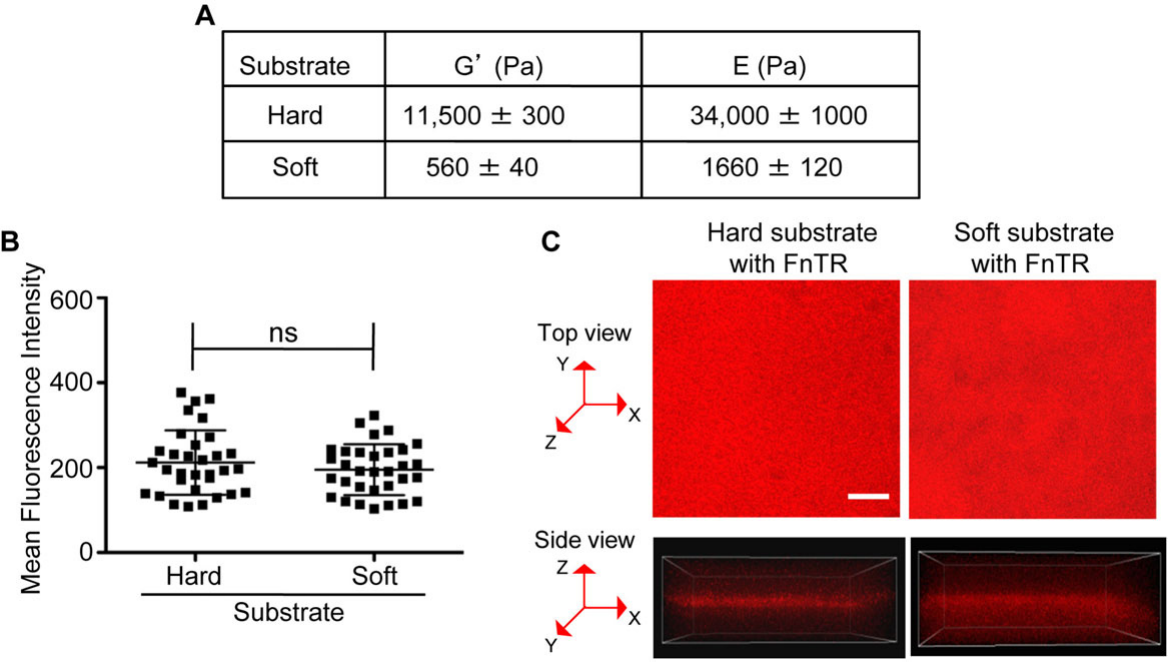
**Compliance and fibronectin coating of hard and soft hydrogels.** (A) The storage moduli (G′) of hard and soft hydrogels was determined as described in Materials and Methods. G′ values were used to calculate the respective Young's moduli (E) as described in Material and Methods. Shown are the mean G′ and E values±s.e.m. from 3 independent experiments. (B,C) The coating density of fibronectin is similar on hard and soft hydrogels. Hard and soft hydrogels were coated with Texas-red conjugated fibronectin (FnTR) as described in Materials and Methods. Confocal images were acquired as z-series spanning the surface of the hydrogels. Four random fields were imaged per hydrogel using identical acquisition parameters. The confocal stacks were then analyzed using Imaris software. On each field, four regions of interest (ROI) with radii of 10.3 µm were drawn at random locations and the mean fluorescent intensity was calculated for each plane of the z-stack for each ROI. (B) Thirty-two ROIs were imaged from two independently prepared hard and soft hydrogels. The plane with the highest mean fluorescence intensity was identified for each ROI; these values are represented by scatter plot. The mean±s.d. are indicated; ns (non-significant). (C) Shown are representative confocal images of FnTR-coated hard and soft substrates from the top view (maximum intensity projected image) and the side view with corresponding x, y and z axes. Bar, 25 µm.

To investigate whether the compliance of the matrix contributes to the integrin-regulation of cytokinesis in HDFs, we used fibronectin-coated acrylamide hydrogels as a model system, as they can be made in a range of physiological stiffness (Klein et al., 2009; Yeung et al., 2005). Dermal fibroblasts adopt a proto-myofibroblast phenotype in culture. Proto-myofibroblasts are proliferative in granulation tissue where compliance has been measured to be E=18,000 Pa (Hinz, 2010). Entry of fibroblasts and epithelial cells into S phase in culture is inhibited on soft matrices (E=2000–4000 Pa) (Bae et al., 2014; Engler et al., 2006; Klein et al., 2009; Pelham and Wang, 1997; Wang et al., 2000; Yeung et al., 2005) and fibronectin matrix assembly is inhibited when fibroblastss are adhered to soft matrices (E=1600 Pa) (Bae et al., 2014; Engler et al., 2006; Klein et al., 2009; Pelham and Wang, 1997; Wang et al., 2000; Yeung et al., 2005). Based on these studies, we made hard and soft hydrogels with stiffnesses of E=34,000 Pa and E=1600, respectively. We measured stiffness by rheology and then used the storage moduli (G′) to calculate the corresponding elastic moduli (E). We were able to reproducibly prepare hard and soft substrates with these compliances (Fig. 2A).

Since adhesion can be affected by fibronectin coating concentration, we examined fibronectin coating on our hard and soft hydrogels. For this purpose, we used Texas-Red-conjugated fibronectin and analyzed fibronectin-coating microscopically. Since the surfaces of hydrogels are uneven, especially compliant hydrogels, we acquired confocal images of hard and soft hydrogels as z-stacks that spanned the adhesive surface of each hydrogel. We identified random regions of interest (ROI) with radii of 10.3 µm and calculated the mean fluorescence intensity for each plane of each ROI and plotted the value for the plane of each ROI with the highest mean fluorescence intensity (Fig. 2B-C). The results indicate that Texas-red fluorescence intensity is similar on hard and soft gels indicating similar levels of fibronectin coating (Fig. 2B-C). Thus, phenotypic differences observed on hard and soft substrates are not due to differences in fibronectin-coating concentrations.

### HDFs require stiff substrates to complete cytokinesis

**Fig. 3.**
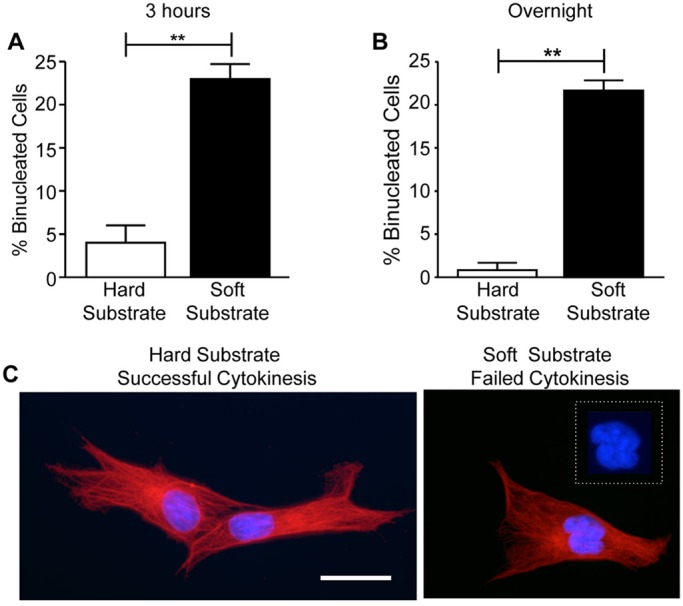
**Matrix compliance regulates cytokinesis in HDFs.** Substrate compliance affects cytokinesis in HDFs. Mitotic HDFs were collected by the shake-off method and replated onto fibronectin-coated hard and soft hydrogels in DMEM with 10% FBS. Cells were incubated for 3 h or overnight at 37°C and then fixed and stained to visualize α-tubulin (red) and nuclei (blue). (A,B) Plotted are the mean percentages of binucleated cells±s.e.m. on hard and soft substrates after either a 3 h (A) or overnight (B) incubation. One hundred cells were counted per condition for each of three independent experiments (**P<0.005). (C) Representative images of cells on hard and soft substrates are shown. Inset: magnified view of nuclei. Bar, 25 µm.

To determine whether substrate compliance affects cytokinesis, mitotic HDFs were collected and replated onto fibronectin-coated hard and soft polyacrylamide hydrogels for 3 h, which is normally sufficient time for HDFs to complete cytokinesis. Cytokinesis was also analyzed following overnight incubation. Cells were fixed, stained and percentage of binucleated cells was determined. The results showed that approximately 23% of mitotic cells fail cytokinesis when adhered to soft substrates (Fig. 3A-C). These results indicate that substrate compliance is a regulator of cytokinesis, and at least in the case of HDFs, stiff substrates promote the successful completion of cytokinesis.

### HDFs are inhibited in abscission on soft substrates

**Fig. 4.**
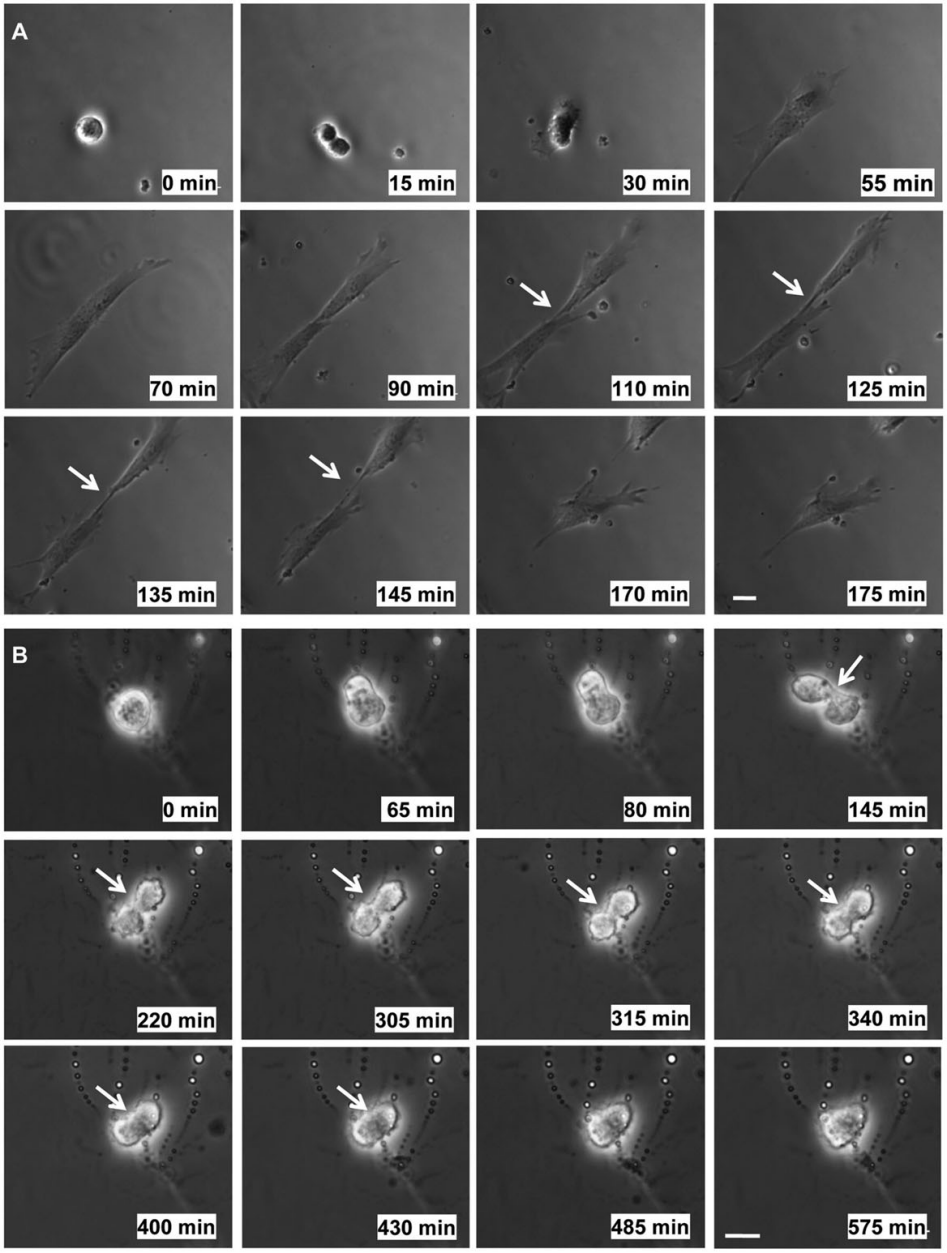
**Time-lapse imaging** **of cytokinesis of HDFs on hard and soft substrates.** Mitotic HDFs were collected by the shake-off method and replated onto fibronectin-coated hard and soft hydrogels in DMEM with 10% FBS. Images were acquired every 5 min for 3–5 h. Individual frames are presented in the montages shown and were chosen to reflect the phenotypic changes (or lack there of) that occur during cytokinesis on hard or soft substrates. The time point of each image is given for each frame. Filming was initiated at time 0. (A) Representative montage of images of a mitotic HDF successfully progressing through cytokinesis resulting in two daughter cells on a hard substrate. (B) Representative montage of images of a mitotic HDF that undergoes ingression of the cleavage furrow, forms a midbody, and subsequently fails cytokinesis on a soft substrate. Bar, 25 µm. Arrows point to the intercellular bridge/midbody and the position where the intercellular bridge is in the process of regressing.

As an alternative approach to monitor cytokinesis, cells were imaged by time-lapse microscopy. Mitotic cells were collected and replated onto hard and soft substrates and imaged as described in Materials and Methods. On hard substrates, mitotic HDFs formed cleavage furrows and midbodies and successfully completed cytokinesis resulting in the separation of two daughter cells (Fig. 4A). Furthermore, HDFs completed cytokinesis within 3 h when mitotic cells were adhered to hard matrices. On soft substrates, mitotic HDFs were able to complete cleavage furrow ingression leaving daughter cells connected by midbodies. Daughter cells remained connected by midbodies for extended periods of time. The midbodies eventually became unstable and presumptive daughter cells fused resulting in failed cytokinesis (Fig. 4B). These results suggest that abscission is inhibited when mitotic HDF are adhered to soft substrates.

**Fig. 5.**
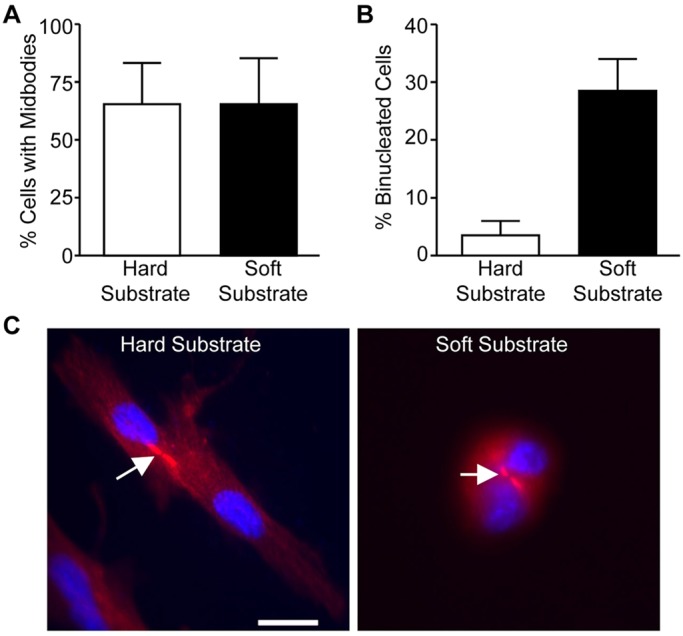
**Mitotic HDFs form midbodies** **on both hard and soft substrates.** Mitotic HDFs were collected by the shake-off method and replated onto fibronectin-coated hard and soft hydrogels in DMEM with 10% FBS and incubated at 37°C for 1.5 h and 3 h. The cells were fixed and stained for α-tubulin (red) and nuclei (blue). (A,B) Plotted is the percentage of cells with midbodies at 1.5 h (A) and that are binucleated at 3 h (B). Data is reported as the mean±the distribution from two independent experiments in which more than fifty cells were counted per condition for each experiment. (C) Representative images of cells at 1.5 h on hard and soft substrates are shown. Bar, 25 µm. Arrows point to the intercellular bridge/midbody.

We next asked whether mitotic HDFs progressed to form midbodies with similar kinetics on soft substrates. Midbody formation was assayed at 1.5 h, as most dividing HDFs are normally connected by midbodies at this time. Midbodies were identified by their distinctive α-tubulin staining (microtubule bundle flanking the midbody ring) (Fig. 5C). The results show that 65% of dividing cells on either hard or soft hydrogels were connected by midbodies at 1.5 h (Fig. 5A). As expected, cells on soft substrates eventually failed cytokinesis (Fig. 5B). These results support the idea that matrix compliance regulates cytokinesis by promoting timely abscission.

### Effect of substrate compliance on cytokinesis is cell-type specific

We were interested to test whether the effect of substrate compliance on cytokinesis is specific to HDFs. For this purpose, we chose C3H 10T1/2 mouse embryonic fibroblasts. These cells are considered mesenchymal stem cells, as they can be triggered to differentiate down several lineages including adipocyte, chondrocyte, skeletal muscle and pericyte lineages (Fu et al., 2013; Pinney and Emerson, 1989; Zhang et al., 2012). Mesenchymal stem cells are of particular interest as their fate is known to be regulated by matrix compliance, with soft matrices promoting neuronal specification, stiffer matrices leading to muscle formation, and rigid matrices resulting in osteoblast differentiation (Engler et al., 2006).

**Fig. 6.**
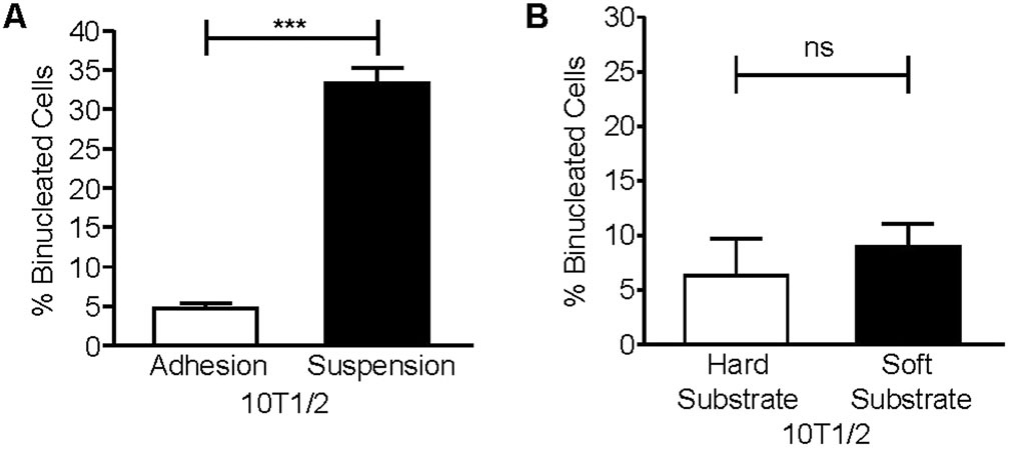
**Embryonic 10T1/2 fibroblasts require cell-matrix adhesion, but not a stiff substrate to complete cytokinesis.** (A) Mitotic 10T1/2 cells were collected and replated onto either fibronectin-coated coverslips (adhesion) or poly-HEMA coated dishes (suspension) at 37°C for 5 h. Cells were stained for α-tubulin and nuclei. Plotted is the mean percentage of binucleated cells±s.e.m. from three independent experiments in which more than one hundred cells were counted per condition for each experiment (***P<0.0005). (B) Mitotic 10T1/2 cells were collected by the shake-off method and replated onto fibronectin-coated hard and soft substrates in DMEM with 10% FBS for 5 h at 37°C and then fixed and stained for α-tubulin, lamin-B1, and nuclei. Plotted is the mean percentage of binucleated cells on hard and soft substrates±s.e.m. from three independent experiments in which one hundred cells were counted per condition for each experiment, ns (non-significant).

Initial experiments tested whether 10T1/2 cells require cell-matrix adhesion to complete cytokinesis. To address this, mitotic 10T1/2 cells were collected and replated for 5 h on fibronectin-coated coverslips or polyHema-coated dishes to prevent adhesion. The percentage of binucleated cells was assayed per condition. The results indicate that cells plated on fibronectin are able to complete cytokinesis. In contrast, 31% of cells that were kept in suspension failed cytokinesis, suggesting that cell-matrix adhesion promotes cytokinesis in 10T1/2 cells (Fig. 6A). We next asked whether matrix compliance regulates cytokinesis in this cell type. We found that 10T1/2 cells successfully completed cytokinesis when adhered to either hard or soft substrates (Fig. 6B). Thus, unlike HDFs, 10T1/2 cells do not require a stiff matrix to successfully complete cytokinesis. Nonetheless, it is possible that cytokinesis in 10T1/2 cells is regulated by matrix compliance, but that much softer matrices are necessary to inhibit cytokinesis. However, our data indicate that the requirement for matrix stiffness is clearly different for HDFs and 10T1/2 cells.

## DISCUSSION

Mechanical properties of the extracellular matrix regulate many cellular processes (DuFort et al., 2011; Schwartz, 2010). In the context of the cell cycle, matrix stiffness has been identified as a key regulator of entry into S phase (Klein et al., 2009; Wang et al., 2000). In our current study, we demonstrate that matrix stiffness also regulates the successful completion of cytokinesis and does so in a cell type-dependent manner. To our knowledge, this is first demonstration that matrix stiffness can promote the successful completion of cytokinesis.

We found that HDFs are able to complete cleavage furrow ingression and form intercellular bridges/midbodies on soft matrices; however, a significant portion of these cells ultimately fail cytokinesis. Time-lapse imaging indicated that on soft substrates dividing HDFs become delayed at the midbody stage remaining connected by intercellular bridges for extended periods of time. It has been proposed that the tractional force exerted by separating daughter cells promotes thinning of the intercellular bridge during late stages of cytokinesis to promote abscission (Burton and Taylor, 1997). We observed thinning of the intercellular bridge when HDFs divided on stiff substrates, whereas on soft substrates thin midbodies were not detected. We showed that β1 integrins promote successful cytokinesis in HDFs. It is known that integrin-mediated adhesion contributes to the development of these traction forces. Others have shown that β1 integrins localize to the edge of lamellopodia at the opposite poles of separating daughter cells, suggesting that new adhesions formed by these protrusions may contribute to the mechanical separation of the daughter cells (Pellinen et al., 2008). Interestingly, β1 integrins also promote the successful completion of cytokinesis in chondrocytes (Aszodi et al., 2003). The role of mechanical signaling in chondrocyte cell division is supported by data from profilin 1-null chondrocytes, which are also inhibited in abscission (Böttcher et al., 2009). Mutant chondrocytes fail to form lamellopodia, to assemble focal adhesions and stress fibers, and to generate strong tractional forces. For these reasons, it was suggested profilin-1 is required for the generation of sufficient force for abscission (Böttcher et al., 2009). Thus, in the case of chondrocytes and HDFs, force-dependent thinning of the intercellular bridge may facilitate abscission. This process may be promoted by alternative mechanisms in other cell types.

Interestingly, a recent study using HeLa cells has shown that pulling exerted on the intercellular bridge by daughter cells as they re-spread actually delayed abscission (Lafaurie-Janvore et al., 2013). Release of this tension triggered the assembly of the ESCRT-III complex followed by membrane scission (Lafaurie-Janvore et al., 2013). Cells on soft substrates exert little tension on their midbodies; however, at least in the case of HDFs, this is not sufficient to promote abscission. It is possible that some cells types may require both tension and its release to successfully complete cytokinesis in timely manner.

Our published studies suggest that integrin signaling may be sufficient to promote cytokinesis at least in CHO cells. CHO cells adhered by recombinant integrins containing a tyrosine to alanine mutation in the membrane-proximal NPIY motif of the integrin β1 cytoplasmic tail are inhibited in spreading, stress fiber formation, ERK activation, and cytokinesis (Aszodi et al., 2003; Kittler et al., 2007; Mathew et al., 2014; Nieves et al., 2010; Pellinen et al., 2008; Reverte et al., 2006; Thullberg et al., 2007). Cytokinesis can be rescued in these cells independent of stress fiber formation by promoting ERK signaling either by activating the mutant integrin with Mn^+2^ or by expressing a constitutively active Raf-1 mutant (Mathew et al., 2014; Nieves et al., 2010). These data suggest that integrin signaling plays an important role in promoting cytokinesis and can do so independent of the development of traction force. Nonetheless, integrin signaling is regulated by matrix stiffness. Studies have shown that matrix stiffness, which facilitates integrin clustering, affects the activation of FAK, ERK, and Rho (Paszek et al., 2005; Provenzano et al., 2009; Wozniak et al., 2003). It is possible that matrix stiffness promotes cytokinesis both by promoting integrin signaling and by allowing separating daughter cells to exert force on the cytoplasmic bridge.

Importantly, not all cell types required a stiff matrix for successful cytokinesis. We found that unlike HDFs, 10T1/2 cells do not require a stiff matrix to successfully complete cytokinesis. However, it is important to note that cytokinesis may be inhibited on much softer matrices than we tested in the studies presented here. Our data also show that 10T1/2 cells require cell-matrix adhesion to promote cytokinesis. This may involve a particular integrin heterodimer or non-integrin adhesion receptors such as syndecans. It will be interesting to understand the nature of the requirement for cell-matrix adhesion and how this relates to the mechanical properties of the substrate. In addition, it will be important to determine whether mesenchymal cells develop a requirement for matrix stiffness for the successful completion of cytokinesis, and if so, the time frame for this requirement after commitment to myoblast or osteoblast lineages, as these processes are promoted by stiff matrices (Engler et al., 2006).

In our experiments, we inhibited β1 integrins as they are the major class of integrin receptors expressed on HDFs and we and others have linked β1 integrins to the regulation of cytokinesis in a number of different cell types (Aszodi et al., 2003; Kittler et al., 2007; Pellinen et al., 2008; Reverte et al., 2006; Thullberg et al., 2007). However, it seems unlikely that the regulation of cytokinesis is restricted to specific β1 integrins or β1 integrins in general. We have shown that chimeric integrins consisting of the extracellular domains of αIIbβ3 connected to the intracellular and transmembrane domains of α5β1 can promote cytokinesis. Others have shown that a significant portion of mitotic cells (40%) adhered to vitronectin, a ligand for β3 and β5 integrins, successfully complete cytokinesis (Thullberg et al., 2007). Clearly, additional in-depth studies are required to understand the role of individual integrins in the regulation of cytokinesis: their contribution may be dictated by expression level, affinity for available ligands, as well as their ability to promote signaling events required for the successful completion of cytokinesis.

In summary, we have identified matrix compliance as a new cell-type specific regulator of cytokinesis. Understanding the cell-type specific contribution of matrix compliance to the regulation of cytokinesis will provide new insights important for our understanding of developmental processes, as well as tissue homeostasis and regeneration.

## MATERIALS AND METHODS

### Cell culture

Human foreskin fibroblasts (HDFs) were purchased from VEC Technologies. C3H/10T1/2, Clone 8 cells (ATCC) were from Dr Mariah Hahn (Rensselaer Polytechnic Institute) and ATCC. Both cell types were cultured in DMEM supplemented with 10% heat-inactivated FBS, 100 units/ml penicillin-streptomycin, and 2 mM L-glutamine. Experiments were performed in supplemented DMEM.

### Cytokinesis assays

Logarithmically growing HDFs or 10T1/2 cells in 150 mm culture dishes were washed with 4 ml of PBS and then 4 ml of supplemented DMEM was added to each dish. Mitotic cells were gently shaken off each dish and then plated at 2×10^5^ cells/35 mm dish onto fibronectin-coated coverslips or hydrogels or incubated in suspension in dishes coated with Poly-HEMA (2-hydroxyethylmethacrylate) (Poly Sciences) as indicated. After 5 h, suspended cells were adhered to fibronectin-coated coverslips and allowed to spread (30 min for HDFs and 15 min for 10T1/2 cells) to facilitate the quantification of the number of binucleated cells, as a measure of failed cytokinesis in suspension, using immunofluorescence microscopy.

### Immunofluorescence microscopy

Cells were fixed with methanol (ice cold, −20°C) for 3 min or 4% formaldehyde for 30 min, permeabilized in 0.5% triton-X-100 for 10 min and blocked with 3% BSA in PBS for 30 min. Cells were then stained with primary antibodies directed against α-tubulin (Santa Cruz Biotechnology, clone: DM1α) and visualized using AlexaFluor568 secondary antibody (Invitrogen). DM1α was used at a final concentration of 0.2 μg/ml and secondary antibodies were used at a final concentration of 2 µg/ml. Nuclei were visualized by wide-field immunoflourescence microscopy by staining with Hoescht 33342 (Invitrogen) at a final concentration of 1 µg/ml or by confocal microscopy using DraQ-5 (Cell Signaling) at a final concentration of 2 µM. In some instances, antibodies to lamin-B1 (Abcam) were used to visualize the nuclear membrane to facilitate the identification of binucleation in poorly spread cells. Antibodies to lamin B1 were used at a final concentration of 1 µg/ml. Fluorescent images were captured using an inverted Nikon TE2000-E microscope equipped with phase contrast and epifluorescence, a digital camera (coolSNAP HQ; Roper Scientific), a Prior ProScanII motorized stage, NIS elements acquisition and analysis software (Nikon) and a C1 confocal system (Nikon) with EZC1 confocal software.

### Time-lapse microscopy

Live-cell imaging was performed at 37°C with 5% CO_2_ in an environmental chamber. For these experiments, mitotic cells were replated on a hydrogel that was tightly held by a circular metallic coverslip holder (Attofluor^®^ Cell Chamber). Mitotic cells were incubated for 30 min in the cell culture incubator to allow them to attach to the substrate. Then the chamber was moved to the microscope stage and images were taken every 5 min for 3 to 5 h.

### Quantification of cytokinesis failure

Cytokinesis failure was quantified by one of three methods depending on the experimental design. (1) When cytokinesis was assayed after mitotic cells were incubated for only 3 h, daughter cells that had successfully completed cytokinesis remained close to each other. This configuration was identified as a successful cytokinesis event. Cytokinesis failure was identified by binucleation. The number of binucleated cells to the total number of cytokinesis events was calculated to determine the percentage of binucleated cells. (2) In the experiments where mitotic cells were incubated for 5 h or overnight, daughter cells were difficult to distinguish from single non-mitotic cells as the cells migrated during these time periods. Hence, the fraction of binucleated cells was calculated by using the following formula, which takes into account the percentage of cells that were mitotic at the time of shake-off, as determined by IF microscopy. Fcyt=Fb(1+Fm)/Fm(1+Fb), where Fcyt was the fraction of mitotic cells that fail cytokinesis, Fm was the fraction of mitotic cells and Fb was fraction of total cells that were binucleated. (3) In the β1 integrin siRNA experiment, cells were treated with siRNA for 72 h, and hence, the percentage of cells that had entered mitosis was not known. In these experiments, the cytokinesis failure was calculated by counting single and binucleated cells.

### β1 siRNA transfection

HDFs were transfected with non-targeting siRNA or 20 nM integrin β1 siRNA using Oligofectamine (Invitrogen) as previously described (Colello et al., 2010). After 72 h, cells were trypsinized and divided into two aliquots. One was lysed with modified RIPA buffer (50 mM Tris-HCl pH 7.4, 1 mM EDTA, 0.25% deoxycholate, 1% NP-40, 150 mM NaCl, 1/100 dilution of each protease inhibitor cocktail (Sigma) and phosphatase inhibitor cocktail (Thermo Scientific) and analyzed by western blotting. The other was plated on coverslips for 6 h and then fixed and stained to assess cytokinesis failure. SiRNAs were from Dharmacon: SiGENOME non-targeting siRNA pool 2 was used as a control. β1 siRNA: sense sequence ACACUGAAUGCAAAGUAGdTdT and antisense sequence CUACUUUGCAUUCAGUGUdTdT.

### Preparation of hard and soft polyacrylamide hydrogels

Polyacrylamide hydrogels were prepared between two coverslips, one reactive and one siliconized, using previously published methods (Cretu et al., 2010; Klein et al., 2007) with the following modifications. The acrylamide concentration was 7.5% for both hard and soft hydrogels; the bis-acrylamide concentration was 0.16% for hard hydrogels and 0.016% for soft hydrogels. To reproducibly generate hard and soft substrates of the desired compliances, hard hydrogels were polymerized for 10 min at 37°C and soft hydrogels were polymerized for 5 min at 37°C. Gelatin (0.3% in PBS) was crosslinked to hydrogels by overnight incubation at 4°C, using acrylic acid N-hydroxy succinimide ester as a crosslinking agent, which was incorparated into the acrylamide mixture as previously described by others (Cretu et al., 2010; Klein et al., 2007). Gelatin-linked hydrogels were then coated with 30 µg/ml human plasma fibronectin in (Millipore) for 1 h at 37°C. Unlabeled fibronectin was from Millipore and Texas-Red labeled fibronection was from Dr Sottile (University of Rochester).

### Rheological measurements

Rheological measurements of the hydrogels were performed with an ARG2 Rheometer using a procedure adapted from a previous study (Zuidema et al., 2014). The storage modulus (G′) of fully formed gels was taken from frequency sweep tests. The thickness of hard and soft gels was measured at three different locations at the center of each gel using a micrometer. The average thickness for hard gels was 600 µm and 800 µm for soft gels. Based on the thickness of the gel, the gap size (distance between lower plate and 8 mm probe) was set for each gel. In order to provide equal indentation on hard and soft substrates, the gap was set so that it was 100 µm less than the gel thickness. Measurements were performed using an 8 mm stainless steel parallel plate at 25°C. Strain sweeps from 0.1–100% strain were used to determine the linear viscoelastic limit of the hydrogels. From these tests, 2% strain was shown to be in the linear viscoelastic limit of the hydrogels. Frequency sweeps at 2% strain were then performed from 0.1 Hz to 100 Hz and the storage modulus corresponding to 1 Hz was recorded, as this region of the curve was within the linear equilibrium modulus plateau of the hydrogels tested. Gels were measured at three different places at the center and an average value was taken. Cellular phenotypes were also analyzed near the center of the hydrogel where compliance measurements were more consistent. Storage moduli (G′) were used to calculate the respective Young's moduli (E) using the formula E=2G′(1+v) where Poisson's ratio (v) for polyacrylamide is 0.48 (Boudou et al., 2006; Carraher and Schwarzbauer, 2013). Soft substrates had calculated Young's moduli of ∼1600 Pa and hard substrates ∼34,000 Pa.

### Analysis of fibronectin-coating on hydrogels

Hydrogels-coated with Texas-Red fibronectin were analyzed by confocal microscopy. Z-stacks with 0.4 µm steps were obtained at four random fields around the center for each hard and soft gel. The confocal stacks were then analyzed using Imaris software (Bitplane). Four random regions of interest (ROI) were chosen per stack using the “contouring” feature in Imaris software. The radius of the each ROI was 10.3 µm. The mean intensity value was calculated for each plane of each ROI and used for analysis.

### Statistical analysis

Statistical analysis was performed using GraphPad Prism software. Student's *t*-test was used where two sets of data were compared. *P*<0.05 indicates statistically significant results.
